# Supplementary material for: Association between psychological distress, lifestyle and career planning on health-related quality of life among the university students during school closure of COVID-19 pandemic in China
Source: PLoS One. 2026 Apr 30;21(4):e0347032. doi: 10.1371/journal.pone.0347032 (PMC13132209; doi:10.1371/journal.pone.0347032)
Supplement: Attachment — Submitted filename: renamed_d24fb.docx [file pone.0347032.s001.docx]

**Revision of ‘PONE-D-25-59759’**

The manuscript “Association Between Psychological Distress, Lifestyle and Career Planning on Health-Related Quality of Life Among the University Students during the COVID-19 Pandemic School Closure in China,” presents original cross-sectional design research examining the determinants of Health-Related Quality of Life (HRQOL) in 1965 Chinese university students during the COVID-19 school closure. I now submit some minor revisions I recommend the authors include, based on PLOS ONE’s criteria.

**Criteria 1 The study presents the results of original research**

The submitted manuscript is a research article reporting findings from a cross-sectional study conducted among university students in China during the COVID-19 school closure period. The authors claim that this work is the first study to explore the determinants of health-related quality of life (HRQOL) among this population from the combined perspective of psychological distress, lifestyle habits, and career planning. The purpose of the study, which is to explore these associations, is clearly stated.

**Criteria 2 Results reported have not been published elsewhere**

The submission is presented as a manuscript draft undergoing the peer review process. Based on the contents, which detail the methodology, novel statistical analyses, and conclusions derived from the collected data of 1965 respondents, the findings appear to be reported here for the first time. However, the authors do not state this explicitly.

**Criteria 3 Experiments statistics and analyses are performed to a high standard and are described in sufficient detail**

The study utilized standard measures, including the SF-12 for HRQOL and the Kessler Psychological disease scale (K10) for psychological distress. The analyses employed Chi-squared tests and binary logistic regression models (Models 1, 2, and 3) to explore associations between variables and HRQOL components (PCS and MCS). The authors performed collinearity diagnostics and confirmed the absence of multicollinearity among independent variables across the models. However, the methodology section notes that due to the lack of a systematic scale, the measurement of lifestyle habits and career planning relied only on selected relevant indicators, which is a noted limitation. Furthermore, the non-random snowball sampling method employed may introduce bias in the sample representativeness.

**Criteria 4 Conclusions are supported by the data and presented appropriately**

The conclusions confirm that low psychological distress, a normal lifestyle, and clearer career planning were associated with higher HRQOL among the university students during the school closure. The presented logistic regression data supports this, showing strong odds ratios linking lower psychological distress to better PCS (OR=3.909) and MCS (OR=8.330) outcomes. Similarly, high physical activity intensity was positively associated with good PCS (OR=4.589). While the data supports the associations presented, the authors correctly identify a limitation inherent to the study design: the cross-sectional nature prevents the prediction of causal relationships.

**Criteria 5 The article is intelligible and written in standard English**

The article is generally intelligible and uses appropriate academic language throughout the Abstract, Methods, Results, and Discussion sections. The authors acknowledge that English language proofreading was conducted during the revision process.

**Criteria 6 The research meets ethical standards and is ethically sound**

The study meets ethical standards as confirmed by the ethics statement. The research design aligned with the tenets of the Declaration of Helsinki, 1996, and received formal approval from the Shandong University Institutional Ethics Committee (Task no. LL20220425). Written informed consent for participation was obtained from the participants’ legal guardians.

**Criteria 7 The article adheres to reporting guidelines and community data availability standards**

The authors have provided necessary declarations regarding funding and competing interests, noting support from the National Natural Science Foundation of China (No. 71804094) and Fundamental Research Funds for the Central Universities (No. 2022KJGL01), and confirming that no competing interests exist. However, the authors state that adherence to community data availability standards is restricted. The data cannot be shared publicly because of concerns involving participant privacy.
