# Supplementary material for: Association between psychological distress, lifestyle and career planning on health-related quality of life among the university students during school closure of COVID-19 pandemic in China
Source: PLoS One. 2026 Apr 30;21(4):e0347032. doi: 10.1371/journal.pone.0347032 (PMC13132209; doi:10.1371/journal.pone.0347032)
Supplement: Attachment — Submitted filename: point by point for plos one 20260207-KSZ.docx [file pone.0347032.s002.docx]

**Point-by-point response to editors and reviewers**

**Authors’ responses to Editor**

Manuscript ID：*PONE-D-25-59759*

Title：Association between Psychological Distress, Lifestyle and Career Planning on Health-Related Quality of Life among the University Students during School Closure of COVID-19 Pandemic in China

Authors：Baochen Su, Zhengna Sun, Rui Chen, Hui Liu, Xixing Xu, Fanlei Kong

The authors appreciate the editor and reviewers’ kind support on reviewing our manuscript and providing us with insightful and detailed comments. We highly value the reviewers’ thoughtful feedback and the important issues highlighted by them. We have addressed each reviewer’s comments in a point-by-point response below. For clarity, we have reproduced the reviewers’ original text in full, with “Q” representing their comments and “A” representing our responses (in red text). Moreover, the authors have highlighted all the modified parts in yellow so that they could be read more clearly by the editor and reviewers.

The authors carefully read the PLOS One guidelines for authors and performed a standardized conversion of the article structure, citations, graphics, and table formats, and invited a native English-speaking editor to grammatically assess this article to ensure compliance with the journal's publication standards.

Below are our point-to-point responses to reviewers' comments:

**Authors’ responses to Reviewer 1**

**Q1：The study presents the results of original research**

The submitted manuscript is a research article reporting findings from a cross-sectional study conducted among university students in China during the COVID-19 school closure period. The authors claim that this work is the first study to explore the determinants of health-related quality of life (HRQOL) among this population from the combined perspective of psychological distress, lifestyle habits, and career planning. The purpose of the study, which is to explore these associations, is clearly stated.

**A1:** Thank you for recognizing the originality and purpose of our study. We extend our heartfelt thanks to your thorough and fundamentally supportive review. Your endorsement of the study’s core contribution, coupled with their precise guidance for improvement, has made the revision process both rewarding and productive. We believe our research will be substantially stronger.

**Q2: Results reported have not been published elsewhere**

The submission is presented as a manuscript draft undergoing the peer review process. Based on the contents, which detail the methodology, novel statistical analyses, and conclusions derived from the collected data of 1965 respondents, the findings appear to be reported here for the first time. However, the authors do not state this explicitly.

**A2**: We are deeply appreciative of the reviewer’s positive remarks about the Originality of the Article. We confirm that the findings presented in this manuscript have not been published elsewhere and are not under consideration for publication in any other journal. We have added a statement to this effect in the cover letter and will include it in the “conflict of Interest” section of the manuscript for clarity.

The corresponding changes could be found on Line 493 – Line 496 in the revised manuscript as follow.

The authors declare that the research was conducted in the absence of any commercial or financial relationships that could be construed as a potential conflict of interest. We confirm that the findings presented in this manuscript have not been published elsewhere and are not under consideration for publication in any other journal.

**Q3: Experiments statistics and analyses are performed to a high standard and are described in sufficient detail**

The study utilized standard measures, including the SF-12 for HRQOL and the Kessler Psychological disease scale (K10) for psychological distress. The analyses employed Chi-squared tests and binary logistic regression models (Models 1, 2, and 3) to explore associations between variables and HRQOL components (PCS and MCS). The authors performed collinearity diagnostics and confirmed the absence of multicollinearity among independent variables across the models. However, the methodology section notes that due to the lack of a systematic scale, the measurement of lifestyle habits and career planning relied only on selected relevant indicators, which is a noted limitation. Furthermore, the non-random snowball sampling method employed may introduce bias in the sample representativeness.

**A3:** We appreciate the reviewer’s acknowledgment of our methodological approach. We have provided a clear description of these limitations in the limitations section of the article, and we will strive to address these issues in future research designs to achieve better results.

**Q4: Conclusions are supported by the data and presented appropriately**

The conclusions confirm that low psychological distress, a normal lifestyle, and clearer career planning were associated with higher HRQOL among the university students during the school closure. The presented logistic regression data supports this, showing strong odds ratios linking lower psychological distress to better PCS (OR=3.909) and MCS (OR=8.330) outcomes. Similarly, high physical activity intensity was positively associated with good PCS (OR=4.589). While the data supports the associations presented, the authors correctly identify a limitation inherent to the study design: the cross-sectional nature prevents the prediction of causal relationships.

**A4:** We sincerely thank the reviewer for their generous appraisal, particularly regarding our section of conclusion. Such recognition motivates us greatly, and we have endeavored to maintain these strengths for longitudinal studies in future research.

**Q5: The article is intelligible and written in standard English**

The article is generally intelligible and uses appropriate academic language throughout the Abstract, Methods, Results, and Discussion sections. The authors acknowledge that English language proofreading was conducted during the revision process.

**A5:** Thank you for your positive feedback regarding the language and readability of our manuscript. We are pleased that the language quality was found acceptable. We acknowledge that English language proofreading was indeed conducted during the revision process to ensure the quality and clarity of the manuscript. We appreciate your recognition of our efforts in this regard.

**Q6: The research meets ethical standards and is ethically sound**

The study meets ethical standards as confirmed by the ethics statement. The research design aligned with the tenets of the Declaration of Helsinki, 1996, and received formal approval from the Shandong University Institutional Ethics Committee (Task no. LL20220425). Written informed consent for participation was obtained from the participants’ legal guardians.

**A6:** Thank you very much for your recognition of the ethical aspects of this research. We confirm that the study was conducted in accordance with the Declaration of Helsinki and approved by the Shandong University Institutional Ethics Committee (Task no. LL20220425). We have explicitly restated this in the Ethics Statement section.

**Q7: The article adheres to reporting guidelines and community data availability standards**

The authors have provided necessary declarations regarding funding and competing interests, noting support from the National Natural Science Foundation of China (No. 71804094) and Fundamental Research Funds for the Central Universities (No. 2022KJGL01), and confirming that no competing interests exist. However, the authors state that adherence to community data availability standards is restricted. The data cannot be shared publicly because of concerns involving participant privacy.

**A7:** We appreciate the comment regarding data availability. We have clarified in the Data Availability Statement that due to ethical and privacy restrictions, the data cannot be shared publicly. However, we confirm that anonymized data is available upon reasonable request from the corresponding author, in compliance with institutional and ethical guidelines.

Once again, thanks very much for all your valuable and insightful comments.

Best wishes.

Fanlei Kong

Corresponding Author

**Authors’ responses to Reviewer 2**

**Q1：**Under the Materials and Methods, there should be exclusion and inclusion criteria to understand the category of group that the study considered. it is also reported that the snowballing sampling method was used in collecting the online data. It should be clearly stated in the manuscript how it was done during the data collection.

**A1:** Thank you for your insightful review of the manuscript and for your helpful comments on this section. This suggestion has been of great help in improving the quality of the manuscript, and the authors attach great importance to it. The authors have supplemented the details of the inclusion and exclusion criteria as well as the sample collection process, with the following specific changes. The revised manuscript is as followed.

The corresponding changes could be found on Line 195 – Line 221 in the revised manuscript as follow.

*2.1. Data and Sample*

Cross-sectional survey design and anonymous online questionnaire were used to assess the quality of life of university students during the school closure of COVID-19. The survey was conducted through an online platform called “Wenjuan.com” (commonly used in China), using a snowball sampling method. Initial respondents were recruited via university student WeChat groups; they were then encouraged to share the questionnaire link with their peers who also met the eligibility criteria, thereby expanding the sample through chain-referral. Data collection took place from May 5th to May 12th, 2022, during the period of school closure. All participants provided informed consent electronically by agreeing to participate before starting the questionnaire.

The snowball sampling method was used due to the difficulty of accessing the nationally dispersed university student population through traditional random sampling during strict campus lockdowns. This approach effectively reached the target population through participants' social networks in the absence of a reliable sampling frame.

Participants were recruited according to the following protocol-defined eligibility requirements: only Chinese students who were officially registered at a mainland university and were obliged to reside on campus under the institution’s COVID-19 “closed-campus” policy were eligible; international students, those living off-campus, and any individuals with a pre-existing major physical or psychiatric disorder that might independently affect the quality of life were excluded.

The minimum sample size was calculated using the cross-sectional formula n = [Z²₁₋α/₂ × P × (1 – P) / d², with Z = 1.96 (95% CI), P = 0.25, d = 0.05 and a design effect of 1.3, yielding 416 participants after 10% invalid allowance. To ensure statistical power and account for invalid responses, after excluding incomplete responses and malicious filling, a total of 1965 questionnaires were used in this study well exceeding the minimum requirement.

**Q2:** Under results, participants' characteristics are incomplete because we are sure of the age group of students you considered in the study and whether they are all nationals or not.

**A2:** Thank you for this helpful comment. The authors highly value your feedback and have made systematic responses and revisions. In response to your concern regarding the completeness of the participants’ characteristics, we have improved the participants' characteristics and revised Table 1 and the results.

1.Age: The overall mean age is 22.3 ± 1.7 years. Because the entire sample consisted of college students enrolled in mainland Chinese universities, this small dispersion reflects normal variation and has no clinical or statistical relevance for the present analyses; therefore, no additional categorization was applied.

2.Nationality: All participants were Chinese nationals holding mainland PRC identification. The following sentence has been added to the Participants subsection: “All students were Chinese studied in Chinese universities.”

3.Region: Table 1 now includes a “Region” variable, showing that 74.9% of participants were recruited from universities in northern China and 25.1% from southern China. Chi-square tests indicated no significant between-region differences for either PCS or MCS (*P* = 0.679 and 0.134, respectively), suggesting that regional distribution did not materially influence the main outcomes.

We believe these additions adequately clarify the homogeneity and representativeness of the sample and address the issue of incomplete participant characteristics.

The corresponding changes could be found on Line 275 – Line 292 in the revised manuscript as follow.

*3.1.* Basic characteristics of the participants

The basic information about the participants was provided in Table 1. All students were Chinese studied in Chinese universities with an average age of 22.25 ± 1.71. It was shown that 59.8% of the sample was female. 55.8% of participants were rural registered residence. As for the region of the participants, 74.9% of participants were recruited from universities in northern China and 25.1% from southern China. Chi-square tests indicated no significant between-region differences for either PCS or MCS (*P* = 0.679 and 0.134)

Table 1 Basic characteristics of the participants

| **Variables** | **Total** | **Good PCS** | **Poor PCS** | ***P*** | **Good MCS** | **Poor MCS** | ***P*** |
| --- | --- | --- | --- | --- | --- | --- | --- |
|  | **n (%)** | **n (%)** | **n (%)** |  | **n (%)** | **n (%)** |  |
| **Age** | 22.25 ± 1.71 | 23.0±1.41 | 21.5±2.12 | 0.801 | 21.8±2.40 | 21.6±2.45 | 0.081 |
| **Gender** |  |  |  | <0.001 |  |  | 0.316 |
| Male | 790(40.2) | 555(70.3) | 235(29.7) |  | 606(76.7) | 184(23.3) |  |
| Female | 1175(59.8) | 943(80.3) | 232(19.7) |  | 898(75.1) | 297(24.9) |  |
| **hukou** |  |  |  | 0.022 |  |  | 0.305 |
| Rural | 1096(55.8) | 814(74.3) | 282(25.7) |  | 818(74.6) | 278(25.4) |  |
| City | 869(44.2) | 684(78.7) | 185(21.3) |  | 666(76.6) | 203(23.4) |  |
| **Region** |  |  |  | 0.679 |  |  | 0.134 |
| northern China | 1472(74,9) | 1119(74.7) | 353(75.6) |  | 1100(74.1) | 372(77.3) |  |
| southern China | 493(25.1) | 379(25.3) | 114(24.4) |  | 384(25.9) | 109(22.7) |  |

Once again, thanks very much for all your valuable and insightful comments.

Best wishes.

Fanlei Kong

Corresponding Author

**Authors’ responses to Reviewer 3**

**Q1:** The abstract should present a clear overview of the study's objectives and the general findings. However, the first paragraph should start with a sentence about the importance of the study and then the purpose of the research.

**A1:** We are very honored to receive your careful review and thank you for your constructive comments. Recognizing the importance of these points, we have undertaken a significant revision of the affected section. We have restructured the abstract as recommended. The first sentence now states the importance of the study, which is directly followed by the purpose of the research.

The corresponding changes could be found on Line 52 – Line 92 in the revised manuscript as follow.

**Abstract:** The COVID-19 pandemic and associated school closures presented unprecedented challenges to university students' well-being, highlighting an urgent need to understand the factors influencing their health-related quality of life. This study aimed to explore the association between psychological distress, lifestyle, and career planning on the health-related quality of life of university students during the school closure period in China. A cross-sectional study was conducted, collecting data from 1965 Chinese college students locked down in campus during COVID-19 using a snowball sampling method via an online questionnaire platform ([Wenjuan.com](https://wenjuan.com/)). Psychological distress and the health-related quality of life were measured by scales. Descriptive analysis, Chi-squared test and logistic regression analysis were employed to analyze the data. The mean physical component summary score was 47.5, while the mean mental component summary score was 36.8. Students with low psychological distress, high physical activity intensity, a regular studying schedule arrangement, clear career planning during COVID-19 were more likely to report a good physical component summary. Conversely, who were women, having a boy or girl friend, with rural hukou were associated with a lower likelihood of good physical component summary. For mental component summary, low psychological distress, a regular studying schedule arrangement and keeping same job-hunting pressure were positive influencing factors, whereas having a boy or girl friend and having no clear career planning during COVID-19 were negative influencing factors. In conclusion, while health-related quality of life was generally good, lower psychological distress, healthier lifestyle habits, and clearer career planning were significantly associated with better health-related quality of life among university students during the pandemic-related school closure. These findings underscore the importance of integrating mental health support, lifestyle guidance, and career planning services into student support systems during public health crises. Future studies should develop and evaluate interventions targeting these modifiable factors.

**Q2:** In the methodology part of the abstract, it is not mentioned about the population and the data gathering tool. Please complete it.

**A2:** We thank the reviewer for carefully identifying this inconsistency. We agree that this information is essential and we have revised the methodology section of the abstract to include a brief description of the study population (1965) and the primary data collection instrument([Wenjuan.com](https://wenjuan.com/)).

The corresponding changes could be found on Line 52 – Line 92 in the revised manuscript as follow.

**Abstract:** The COVID-19 pandemic and associated school closures presented unprecedented challenges to university students' well-being, highlighting an urgent need to understand the factors influencing their health-related quality of life. This study aimed to explore the association between psychological distress, lifestyle, and career planning on the health-related quality of life of university students during the school closure period in China. A cross-sectional study was conducted, collecting data from 1965 Chinese college students locked down in campus during COVID-19 using a snowball sampling method via an online questionnaire platform ([Wenjuan.com](https://wenjuan.com/)). Psychological distress and the health-related quality of life were measured by scales. Descriptive analysis, Chi-squared test and logistic regression analysis were employed to analyze the data. The mean physical component summary score was 47.5, while the mean mental component summary score was 36.8. Students with low psychological distress, high physical activity intensity, a regular studying schedule arrangement, clear career planning during COVID-19 were more likely to report a good physical component summary. Conversely, who were women, having a boy or girl friend, with rural hukou were associated with a lower likelihood of good physical component summary. For mental component summary, low psychological distress, a regular studying schedule arrangement and keeping same job-hunting pressure were positive influencing factors, whereas having a boy or girl friend and having no clear career planning during COVID-19 were negative influencing factors. In conclusion, while health-related quality of life was generally good, lower psychological distress, healthier lifestyle habits, and clearer career planning were significantly associated with better health-related quality of life among university students during the pandemic-related school closure. These findings underscore the importance of integrating mental health support, lifestyle guidance, and career planning services into student support systems during public health crises. Future studies should develop and evaluate interventions targeting these modifiable factors.

**Q3:** In the result part of the abstract, the authors should bring the complete words instead of the acronyms they have used (MCS = mental component summary, PCS = physical component summary)

**A3:** We thank the reviewer for pointing out this oversight. We have corrected it in the revised version. It was an error on our part, and we have corrected it accordingly in the revised version. We have replaced the acronyms "MCS" and "PCS" with their full terms, "mental component summary" and "physical component summary," respectively, throughout the abstract. The acronyms are now only introduced and defined in the main text.

The corresponding changes could be found on Line 52 – Line 92 in the revised manuscript as follow.

**Abstract:** The COVID-19 pandemic and associated school closures presented unprecedented challenges to university students' well-being, highlighting an urgent need to understand the factors influencing their health-related quality of life. This study aimed to explore the association between psychological distress, lifestyle, and career planning on the health-related quality of life of university students during the school closure period in China. A cross-sectional study was conducted, collecting data from 1965 Chinese college students locked down in campus during COVID-19 using a snowball sampling method via an online questionnaire platform ([Wenjuan.com](https://wenjuan.com/)). Psychological distress and the health-related quality of life were measured by scales. Descriptive analysis, Chi-squared test and logistic regression analysis were employed to analyze the data. The mean physical component summary score was 47.5, while the mean mental component summary score was 36.8. Students with low psychological distress, high physical activity intensity, a regular studying schedule arrangement, clear career planning during COVID-19 were more likely to report a good physical component summary. Conversely, who were women, having a boy or girl friend, with rural hukou were associated with a lower likelihood of good physical component summary. For mental component summary, low psychological distress, a regular studying schedule arrangement and keeping same job-hunting pressure were positive influencing factors, whereas having a boy or girl friend and having no clear career planning during COVID-19 were negative influencing factors. In conclusion, while health-related quality of life was generally good, lower psychological distress, healthier lifestyle habits, and clearer career planning were significantly associated with better health-related quality of life among university students during the pandemic-related school closure. These findings underscore the importance of integrating mental health support, lifestyle guidance, and career planning services into student support systems during public health crises. Future studies should develop and evaluate interventions targeting these modifiable factors.

**Q4:** Some claims and sentences do not have references. For instance, lines 119-121-47. Please check the article.

**A4:** We apologize for these omissions. We have thoroughly reviewed the entire manuscript and added appropriate citations to support all claims that required references, including those on lines 47, 119, and 121. The reference list has been updated accordingly. Reference 4, 5, 6, 8 and 9 were newly added in the manuscript.

The corresponding changes could be found on Line 162 – Line 173 in the revised manuscript as follow.

Lifestyle habits has been proven to be one of the most important issues affecting the physical and mental health of the students.[1] A German study found that University students who had better sleep tend to had better HRQOL,[2] while the adolescents with regular sports activity would had higher HRQOL during stay at home than those with non-regular sports activity.[3] Previous research on lifestyle habits has focused on smoking and drinking which are relatively rare among the university students while the physical activity[4] and sleep habits[5] are very common among the university students which changed a lot during the lockdown period and have a significant impact on their lives.

Literature reviews showed there were few research on the relationship between career planning and HRQOL among the university students currently.[6] A previous study found that there was a significant difference on the student career perception before and during the COVID-19,[7] while the changes in the student career perception will bring the changes in their career planning and even career pressure.[8, 9] Thus, the school closure of the COVID-19 may affect the students’ career planning.

**Q5:** The "Introduction" should be developed, modified, rearranged, and divided into some paragraphs based on scientific writing. To strengthen the paper, I would suggest expanding the introduction and bringing more from the literature about the idea of this paper; that would make a stronger contribution to the international audience.
**A5:** This is indeed an important consideration. To make this more explicit for readers, we have now comprehensively revised the Introduction by expanding the literature review, restructuring the content into logically flowing paragraphs, and strengthening the theoretical framework to better contextualize our research and enhance its relevance for an international audience.

The corresponding changes could be found on Line 93 – Line 192 in the revised manuscript as follow.

1. Introduction

Since December 2019, the novel coronavirus disease 2019 (COVID-19) has spread from Wuhan city to different areas in China and around the world.[10] As of December 2022, there are 9311491 Chinese people had been infected with COVID-19.[11] This pandemic has disrupted our normal pace of life, and furtherly brought many health problems.[12] No reliable COVID-19-specific therapeutics had been identified yet,[13] which made almost all universities in China adopted the closure policy to ensure the health of university students. School closure is an important way for many Chinese universities in response to the COVID-19. During the semester, all students' studies and lives were on campus, and they can't enter or leave the campus unless they need medical treatment. Many gathering activities were canceled, and many courses were changed to online learning in the dormitories.[14] This closed and repressive environment and the reduction of collective activities will bring some mental and physical problems.[15]

Campus lockdowns abruptly removed the structured social, physical and academic environment that normally sustains student health.[16] While school closures during the COVID-19 pandemic reduced infection risks, they simultaneously triggered a cascade of challenges for university students including academic procrastination, deteriorating mental and physical health, diminished social support, heightened loneliness, and altered Health-Related Quality of Life (HRQOL) [17-19] And school closure may make the situation worse.[20]However, most evidence is based on convenience samples collected during the first semester of the pandemic; little is known about the sustained impact of strict “closed-campus” policies—unique to mainland China—where students were physically confined to dormitories for entire academic years. Consequently, the health status, the quality of life of the university students and its influencing factors during the school closure of the COVID-19 was worth further research.

Health-related quality of life (HRQOL) is defined as an individual’s satisfaction or happiness with the quality of their physical and psychological distress and is deemed a comprehensive assessment tool of a population’s health status.[21] Previous research found that adolescents with internalizing and externalizing psychological distress problems had the lowest HRQOL.[22] University students with good healthy lifestyles may had better HRQOL.[23] While those who had a boy or girl friend, female and physical activity tended to a positive effect on the HRQOL.[24] However the COVID-19 pandemic made these relationships different and more complex.[25] COVID-19 as a public health emergency has been proved by researches that it may affect many aspects of university students, such as mental health [26, 27]and physical health problems.[28]

Demographic factors have always been important influencing factors on HRQOL, and even some studies focus on it as an independent and important influencing factor.[29] Previous study has shown that gender differences have significant impact on the quality of life of medical students.[30] With respect to the medical and psychological aspects, one of the most eminent and influential Persian philosophers and scientists Avicenna held that lovesickness brings disease to the soul and body of a lover.[31] In addition, many basic scientific studies have shown a very close relationship between love and health.[32] So it will be very interesting to explore the association between love and the HRQOL of university students in the special situation where many couples cannot see each other for a long time during the school closure of COVID-19. A Chinese study found that university students who have the urban hukou (registration) enjoyed higher subjective social status, which had a clear protective effect against anxiety and depression symptoms.[33] And another study from China showed that the non-only children were more likely to develop the symptoms of anxiety and depression than only children, during the outbreak of COVID-19 in China.[34]

~~Health-related quality of life (HRQOL) is defined as an individual’s satisfaction or happiness with the quality of their physical and psychological distress and is deemed a comprehensive assessment tool of a population’s health status. (21) Previous research found that adolescents with internalizing and externalizing psychological distress problems had the lowest HRQOL.(22) University students with good healthy lifestyles may had better HRQOL.(23) While those who had a boy or girl friend, female and physical activity tended to a positive effect on the HRQOL.(24) However the COVID-19 pandemic made these relationships different and more complex.(25) COVID-19 as a public health emergency has been proved by researches that it may affect many aspects of university students, such as mental health (26, 27)and physical health problems.(28)~~

Lifestyle habits has been proven to be one of the most important issues affecting the physical and mental health of the students.[1] A German study found that University students who had better sleep tend to had better HRQOL,[2] while the adolescents with regular sports activity would had higher HRQOL during stay at home than those with non-regular sports activity.[3] Previous research on lifestyle habits has focused on smoking and drinking which are relatively rare among the university students while the physical activity[4] and sleep habits[5] are very common among the university students which changed a lot during the lockdown period and have a significant impact on their lives.

Literature reviews showed there were few research on the relationship between career planning and HRQOL among the university students currently.[6] A previous study found that there was a significant difference on the student career perception before and during the COVID-19,[7] while the changes in the student career perception will bring the changes in their career planning and even career pressure.[8, 9] Thus, the school closure of the COVID-19 may affect the students’ career planning.

To conclude, some researches focused on the groups of university students during COVID-19.[35, 36] Few researches focused on gender differences on the quality of life during the COVID-19 pandemic.[37] Only one study had explored the relationship between career planning and HRQOL during the COVID-19 pandemic.[7] Let alone examined the association between psychological distress, lifestyle, and career planning on HRQOL among the university students especially during the school closure of COVID-19. Thus, this study aimed to clarify the association between psychological distress, lifestyle and career planning on HRQOL of the university students during the school closure of COVID-19.

**Q6:** The authors should justify why they have used snowball sampling in this study and mention the population size and how they calculated the sample size in the methodology.

**A6:** Thank you for your advice and for prompting us to clarify our methodology. The authors had added the platform for data collection and appreciate your suggestion on the sample collection.

The research time in this study was during the school closure of COVID-19. Due to the strict policy and the psychological and living conditions of college students during COVID-19, it was impossible to sample the designated group during this period.

Snowball sampling has its unique advantages. Firstly, it is recommended by acquaintances, which can ensure the privacy of respondents and reduce their psychological defense for sensitive topics, thus ensuring the quality of questionnaire responses. Secondly, it can collect the maximum number of questionnaires at a low cost over a long period of time. We cannot control the duration of the school closure, so this has increased the number of questionnaires and greatly increased the probability of completing this study. Therefore, in summary, using snowball sampling during this special period and research has a relatively better impact on both the quantity and quality of questionnaires. Therefore, this study ultimately chose snowball sampling.

Of course, snowball sampling has its natural disadvantage——bias in the representativeness of the sample, which have explained in detail in the “limitations” section of this article.

The minimum sample size was calculated using the cross-sectional formula n = [Z²₁₋α/₂ × P × (1 – P) / d², with Z = 1.96 (95% CI), d = 0.05 and a design effect of 1.3. In this study, we defined poor HRQOL as scores lower than that of the first quartile, so the *P* value is 0.25 in the formula. After calculation and adding 10% invalid allowance, the lowest sample size obtained in this study is 416. The sample size we ultimately collected for analysis (1965) far exceeded 416.

Finally, the authors added three complements in the revised " Data and Sample " section:

A justification for using snowball sampling, citing the challenges in accessing our target population through traditional random sampling methods.

An estimate of the total target population size from which our sample was drawn.

The formula and parameters used for the sample size calculation.

The corresponding changes could be found on Line 194 –Line 211 in the revised manuscript as follow.

*2.1. Data and Sample*

Cross-sectional survey design and anonymous online questionnaire were used to assess the quality of life of university students during the school closure of COVID-19. The survey was conducted through an online platform called “Wenjuan.com” (commonly used in China), using a snowball sampling method. Initial respondents were recruited via university student WeChat groups; they were then encouraged to share the questionnaire link with their peers who also met the eligibility criteria, thereby expanding the sample through chain-referral. Data collection took place from May 5th to May 12th, 2022, during the period of school closure. All participants provided informed consent electronically by agreeing to participate before starting the questionnaire.

The snowball sampling method was used due to the difficulty of accessing the nationally dispersed university student population through traditional random sampling during strict campus lockdowns. This approach effectively reached the target population through participants' social networks in the absence of a reliable sampling frame.

Participants were recruited according to the following protocol-defined eligibility requirements: only Chinese students who were officially registered at a mainland university and were obliged to reside on campus under the institution’s COVID-19 “closed-campus” policy were eligible; international students, those living off-campus, and any individuals with a pre-existing major physical or psychiatric disorder that might independently affect the quality of life were excluded.

The minimum sample size was calculated using the cross-sectional formula n = [Z²₁₋α/₂ × *P* × (1 – *P*) / d², with Z = 1.96 (95% CI), *P* = 0.25, d = 0.05 and a design effect of 1.3, yielding 416 participants after 10% invalid allowance. To ensure statistical power and account for invalid responses, after excluding incomplete responses and malicious filling, a total of 1965 questionnaires were used in this study well exceeding the minimum requirement.

**Q7:** For better readability, the authors should divide the very long table into some specific and concise tables in the article.

**A7:** Thank you for your valuable advice, we have divided table 1 into two tables.

Tables 2 and 3 present a hierarchical (three-step) binary logistic regression analysis that is conceptually and analytically an integral set; separating them would fragment the logical flow of the models. Consequently, these two tables have been retained as they are.

In contrast, the original Table 1 was markedly long. In response to your comment, we have now split it into two distinct but consecutive tables (Table 1A and Table 1B) to improve readability while preserving all the descriptive data.

The corresponding changes could be found on Line 293 –Line 298 in the revised manuscript as follow.

Table 1 Basic characteristics of the participants

| **Variables** | **Total** | **Good PCS** | **Poor PCS** | ***P*** | **Good MCS** | **Poor MCS** | ***P*** |
| --- | --- | --- | --- | --- | --- | --- | --- |
|  | **n (%)** | **n (%)** | **n (%)** |  | **n (%)** | **n (%)** |  |
| **Age** | 22.25 ± 1.71 | 23.0±1.41 | 21.5±2.12 | 0.810 | 21.8±2.40 | 21.6±2.45 | 0.081 |
| **Region** |  |  |  | 0.679 |  |  | 0.134 |
| north of China | 1472(74,9) | 1119(74.7) | 353(75.6) |  | 1100(74.1) | 372(77.3) |  |
| south of China | 493(25.1) | 379(25.3) | 114(24.4) |  | 384(25.9) | 109(22.7) |  |
| **Gender** |  |  |  | <0.001 |  |  | 0.316 |
| Male | 790(40.2) | 555(70.3) | 235(29.7) |  | 606(76.7) | 184(23.3) |  |
| Female | 1175(59.8) | 943(80.3) | 232(19.7) |  | 898(75.1) | 297(24.9) |  |
| **hukou** |  |  |  | 0.022 |  |  | 0.305 |
| Rural | 1096(55.8) | 814(74.3) | 282(25.7) |  | 818(74.6) | 278(25.4) |  |
| City | 869(44.2) | 684(78.7) | 185(21.3) |  | 666(76.6) | 203(23.4) |  |

Notes: MCS = mental component summary, PCS = physical component summary.

Table 2 Descriptive for HRQOL in the mental and physical dimensions of the participants

| **Variables** | **Total** | **Good PCS** | **Poor PCS** | ***P*** | **Good MCS** | **Poor MCS** | ***P*** |
| --- | --- | --- | --- | --- | --- | --- | --- |
|  | **n (%)** | **n (%)** | **n (%)** |  | **n (%)** | **n (%)** |  |
| **One child only** |  |  |  | 0.039 |  |  | 0.819 |
| Yes | 845(43.0) | 625(74.0) | 220(26.0) |  | 636(75.3) | 209(24.7) |  |
| No | 1120(57.0) | 873(77.9) | 247(22.1) |  | 848(75.7) | 272(24.3) |  |
| **Love** |  |  |  | <0.001 |  |  | 0.015 |
| Be in love | 899(45.8) | 644(71.6) | 255(28.4) |  | 702(78.1) | 197(21.9) |  |
| Not in love | 1066(54.2) | 854(80.1) | 212(19.9) |  | 782(73.4) | 284(26.6) |  |
| **Psychological distress** |  |  |  | <0.001 |  |  | <0.001 |
| Lower psychological distress | 948(48.2) | 823(86.8) | 125(13.2) |  | 851(89.8) | 97(10.2) |  |
| Low psychological distress | 470(23.9) | 361(76.8) | 109(23.2) |  | 345(73.4) | 125(26.6) |  |
| High psychological distress | 289(14.7) | 166(57.4) | 123(42.6) |  | 174(60.2) | 115(39.8) |  |
| Higher psychological distress | 258(13.1) | 148(57.4) | 110(42.6) |  | 114(44.2) | 144(55.8) |  |
| **Studying schedule arrangement** |  |  |  | <0.001 |  |  | <0.001 |
| Quiet Regular | 378(19.2) | 301(79.6) | 77(20.4) |  | 324(85.7) | 54(14.3) |  |
| Regular most of the time | 1067(54.3) | 851(79.8) | 216(20.2) |  | 848(79.5) | 219(20.5) |  |
| Not very regular | 457(23.3) | 309(67.6) | 148(32.4) |  | 286(62.6) | 171(37.4) |  |
| Not regular | 63(3.2) | 37(58.7) | 26(41.3) |  | 26(41.3) | 37(58.7) |  |
| **Physical activity intensity** |  |  |  | <0.001 |  |  | 0.234 |
| Small amount | 1411(71.8) | 1011(71.7) | 400(28.3) |  | 1051(74.5) | 360(25.5) |  |
| Medium amount | 366(18.6) | 316(86.3) | 50(13.7) |  | 286(78.1) | 80(21.9) |  |
| Large amount | 188(9.6) | 171(91.0) | 17(9.0) |  | 147(78.2) | 41(21.8) |  |
| **Clear career planning** |  |  |  | 0.001 |  |  | <0.001 |
| Yes | 1270(64.6) | 937(73.8) | 333(26.2) |  | 1002(78.9) | 268(21.1) |  |
| No | 695(35.4) | 561(80.7) | 134(19.3) |  | 482(69.4) | 213(30.6) |  |
| **The change of career planning during COVID-19** |  |  |  | <0.001 |  |  | 0.249 |
| Yes | 753(38.3) | 487(64.7) | 266(35.3) |  | 558(74.1) | 195(25.9) |  |
| No | 1212(61.7) | 1011(83.4) | 201(16.6) |  | 926(76.4) | 286(23.6) |  |
| **Job-hunting pressure** |  |  |  | <0.001 |  |  | <0.001 |
| Not at all | 135(6.9) | 105(77.8) | 30(22.2) |  | 110(81.5) | 25(18.5) |  |
| A little | 361(18.4) | 255(70.6) | 106(29.4) |  | 289(80.1) | 72(19.9) |  |
| Some | 1097(55.8) | 880(80.2) | 217(19.8) |  | 858(78.2) | 239(21.8) |  |
| Quite a lot | 309(15.7) | 218(70.6) | 91(29.4) |  | 195(63.1) | 114(36.9) |  |
| Huge | 63(3.2) | 40(63.5) | 23(36.5) |  | 32(50.8) | 31(49.2) |  |
| **The Changes of job-hunting pressure during the school closure of the COVID-19** |  |  |  | 0.014 |  |  | <0.001 |
| Become much lower | 33(1.7) | 28(84.8) | 5(15.2) |  | 29(87.9) | 4(12.1) |  |
| Become lower | 112(5.7) | 77(68.8) | 35(31.3) |  | 82(73.2) | 30(26.8) |  |
| No change | 668(34.0) | 531(79.5) | 137(20.5) |  | 545(81.6) | 123(18.4) |  |
| Become higher | 915(46.6) | 694(75.8) | 221(24.2) |  | 679(74.2) | 236(25.8) |  |
| Become much higher | 237(12.1) | 168(70.9) | 69(29.1) |  | 149(62.9) | 88(37.1) |  |

Notes: HRQOL = health-related quality of life, MCS = mental component summary, PCS = physical component summary.

**Q8:** There are some mistakes, so the language and punctuation of the manuscript need to be revised and improved throughout the text, and the manuscript should be revised by a native English speaker.

**A8:** Thank you for prompting us to consider these points. We have taken this comment seriously. The manuscript has undergone professional language editing by adding a native English-speaking international student who has been thanked at the section of “Acknowledgments”.

The corresponding changes could be found on Line 486 –Line 490 in the revised manuscript as follow.

**Acknowledgments**

The research team greatly appreciates the funding support and the research participants for their cooperation and support. The authors are grateful that Insha Yousuf Ellahie and Tawhida Mwinyi Tallib conducted the English language proofreading of the revised manuscript; Jiajia Li and Shixue Li gave many valuable suggestions on the revision of manuscript during the peer review procedure.

This process has corrected grammatical errors, improved sentence structure, refined word choice, and ensured proper punctuation throughout the text. There have been many changes made, and due to space constraints, we will only display the information on the modified lines below. The corresponding changes could be found on Line 97, Line 97-98, Line 133, Line 169, Line 348, Line 354, Line 355, Line 374, Line 475 in the revised manuscript as follow.

1.	Qin Z, Wang N, Ware RS, Sha Y, Xu F. Lifestyle-related behaviors and health-related quality of life among children and adolescents in China. Health Qual Life Outcomes. 2021;19(1):8. Epub 2021/01/08. doi: 10.1186/s12955-020-01657-w. PubMed PMID: 33407589; PubMed Central PMCID: PMCPMC7788787.

2.	Friedrich A, Claßen M, Schlarb AA. Sleep better, feel better? Effects of a CBT-I and HT-I sleep training on mental health, quality of life and stress coping in university students: a randomized pilot controlled trial. BMC psychiatry. 2018;18(1):268. Epub 2018/08/31. doi: 10.1186/s12888-018-1860-2. PubMed PMID: 30157894; PubMed Central PMCID: PMCPMC6114783.

3.	Zehnder C, Nigg CR, Benzing V. COVID-19: Sports activity and health-related quality of life of Swiss children and adolescents before and during the initial stay at home period. Journal of health psychology. 2022:13591053221122722. Epub 2022/09/10. doi: 10.1177/13591053221122722. PubMed PMID: 36082434; PubMed Central PMCID: PMCPMC9459366.

4.	Romero-Blanco C, Rodríguez-Almagro J, Onieva-Zafra MD, Parra-Fernández ML, Prado-Laguna MDC, Hernández-Martínez A. Physical Activity and Sedentary Lifestyle in University Students: Changes during Confinement Due to the COVID-19 Pandemic. Int J Environ Res Public Health. 2020;17(18). Epub 20200909. doi: 10.3390/ijerph17186567. PubMed PMID: 32916972; PubMed Central PMCID: PMCPMC7558021.

5.	Romero-Blanco C, Rodríguez-Almagro J, Onieva-Zafra MD, Parra-Fernández ML, Prado-Laguna MDC, Hernández-Martínez A. Sleep Pattern Changes in Nursing Students during the COVID-19 Lockdown. Int J Environ Res Public Health. 2020;17(14). Epub 20200720. doi: 10.3390/ijerph17145222. PubMed PMID: 32698343; PubMed Central PMCID: PMCPMC7400502.

6.	Rashid HH, Ghotane SG, Abufanas SH, Gallagher JE. Short and long-term career plans of final year dental students in the United Arab Emirates. BMC Oral Health. 2013;13:39. Epub 20130813. doi: 10.1186/1472-6831-13-39. PubMed PMID: 23937862; PubMed Central PMCID: PMCPMC3751876.

7.	Benaraba CMD, Bulaon NJB, Escosio SMD, Narvaez AHG, Suinan ANA, Roma MN. A Comparative Analysis on the Career Perceptions of Tourism Management Students Before and During the COVID-19 Pandemic. Journal of hospitality, leisure, sport & tourism education. 2022;30:100361. Epub 2021/11/30. doi: 10.1016/j.jhlste.2021.100361. PubMed PMID: 34840530; PubMed Central PMCID: PMCPMC8606300.

8.	Zhang Z, Yu X, Liu X. Do I decide my career? Linking career stress, career exploration, and future work self to career planning or indecision. Front Psychol. 2022;13:997984. Epub 20220823. doi: 10.3389/fpsyg.2022.997984. PubMed PMID: 36081730; PubMed Central PMCID: PMCPMC9448360.

9.	Yilmaz AA, Ilce A, Can Cicek S, Yuzden GE, Yigit U. The effect of a career activity on the students' perception of the nursing profession and their career plan: A single-group experimental study. Nurse Educ Today. 2016;39:176-80. Epub 20160222. doi: 10.1016/j.nedt.2016.02.001. PubMed PMID: 27006053.

10.	Dhama K, Khan S, Tiwari R, Sircar S, Bhat S, Malik YS, et al. Coronavirus Disease 2019-COVID-19. Clinical microbiology reviews. 2020;33(4). Epub 2020/06/26. doi: 10.1128/cmr.00028-20. PubMed PMID: 32580969; PubMed Central PMCID: PMCPMC7405836.

11.	National pamhc. Real time big data report of epidemic situation. 2022. doi: <https://voice.baidu.com/act/newpneumonia/newpneumonia/?from=osari_aladin_banner>.

12.	Petersen JA, Naish C, Ghoneim D, Cabaj JL, Doyle-Baker PK, McCormack GR. Impact of the COVID-19 Pandemic on Physical Activity and Sedentary Behaviour: A Qualitative Study in a Canadian City. Int J Environ Res Public Health. 2021;18(9). Epub 2021/05/01. doi: 10.3390/ijerph18094441. PubMed PMID: 33922094; PubMed Central PMCID: PMCPMC8122654.

13.	Stawicki SP, Jeanmonod R, Miller AC, Paladino L, Gaieski DF, Yaffee AQ, et al. The 2019-2020 Novel Coronavirus (Severe Acute Respiratory Syndrome Coronavirus 2) Pandemic: A Joint American College of Academic International Medicine-World Academic Council of Emergency Medicine Multidisciplinary COVID-19 Working Group Consensus Paper. Journal of global infectious diseases. 2020;12(2):47-93. Epub 2020/08/11. doi: 10.4103/jgid.jgid_86_20. PubMed PMID: 32773996; PubMed Central PMCID: PMCPMC7384689.

14.	Bozgun K, Ozaskin-Arslan AG, Ulucinar-Sagir S. COVID-19 and Distance Education: Evaluation in the Context of Twenty-first Century Skills. The Asia-Pacific Education Researcher. 2022. doi: 10.1007/s40299-022-00663-4.

15.	Usher K, Durkin J, Bhullar N. The COVID-19 pandemic and mental health impacts. International journal of mental health nursing. 2020;29(3):315-8. Epub 2020/04/12. doi: 10.1111/inm.12726. PubMed PMID: 32277578; PubMed Central PMCID: PMCPMC7262128.

16.	Sahu P. Closure of Universities Due to Coronavirus Disease 2019 (COVID-19): Impact on Education and Mental Health of Students and Academic Staff. Cureus. 2020;12(4):e7541. Epub 20200404. doi: 10.7759/cureus.7541. PubMed PMID: 32377489; PubMed Central PMCID: PMCPMC7198094.

17.	Auger KA, Shah SS, Richardson T, Hartley D, Hall M, Warniment A, et al. Association Between Statewide School Closure and COVID-19 Incidence and Mortality in the US. Jama. 2020;324(9):859-70. Epub 2020/08/04. doi: 10.1001/jama.2020.14348. PubMed PMID: 32745200; PubMed Central PMCID: PMCPMC7391181.

18.	Albursan IS, Al Qudah MF, Al-Barashdi HS, Bakhiet SF, Darandari E, Al-Asqah SS, et al. Smartphone Addiction among University Students in Light of the COVID-19 Pandemic: Prevalence, Relationship to Academic Procrastination, Quality of Life, Gender and Educational Stage. Int J Environ Res Public Health. 2022;19(16). Epub 2022/08/27. doi: 10.3390/ijerph191610439. PubMed PMID: 36012073; PubMed Central PMCID: PMCPMC9408323.

19.	Pineda CN, Naz MP, Ortiz A, Ouano EL, Padua NP, Paronable JJ, et al. Resilience, Social Support, Loneliness and Quality of Life during COVID-19 Pandemic: A Structural Equation Model. Nurse education in practice. 2022;64:103419. Epub 2022/08/12. doi: 10.1016/j.nepr.2022.103419. PubMed PMID: 35952471; PubMed Central PMCID: PMCPMC9301165.

20.	Aqeel M, Rehna T, Shuja KH, Abbas J. Comparison of Students' Mental Wellbeing, Anxiety, Depression, and Quality of Life During COVID-19's Full and Partial (Smart) Lockdowns: A Follow-Up Study at a 5-Month Interval. Frontiers in psychiatry. 2022;13:835585. Epub 2022/05/10. doi: 10.3389/fpsyt.2022.835585. PubMed PMID: 35530024; PubMed Central PMCID: PMCPMC9067378.

21.	Wang P, Chen C, Yang R, Wu Y. Psychometric evaluation of health related quality of life among rural-to-urban migrants in China. Health Qual Life Outcomes. 2015;13:155. Epub 2015/09/25. doi: 10.1186/s12955-015-0350-1. PubMed PMID: 26399311; PubMed Central PMCID: PMCPMC4581414.

22.	Hunduma G, Dessie Y, Geda B, Yadeta TA, Deyessa N. Internalizing and externalizing mental health problems affect in-school adolescent's health-related quality of life in eastern Ethiopia: A cross-sectional study. PloS one. 2022;17(8):e0272651. Epub 2022/08/05. doi: 10.1371/journal.pone.0272651. PubMed PMID: 35925999; PubMed Central PMCID: PMCPMC9352091.

23.	Li S, Cui G, Zhou F, Liu S, Guo Y, Yin Y, et al. The Longitudinal Relationship Between eHealth Literacy, Health-Promoting Lifestyles, and Health-Related Quality of Life Among College Students: A Cross-Lagged Analysis. Frontiers in public health. 2022;10:868279. Epub 2022/07/26. doi: 10.3389/fpubh.2022.868279. PubMed PMID: 35875048; PubMed Central PMCID: PMCPMC9304771.

24.	Kotarska K, Paczyńska-Jędrycka M, Sygit K, Kmieć K, Czerw A, Nowak MA. Physical Activity and the Quality of Life of Female Students of Universities in Poland. Int J Environ Res Public Health. 2021;18(10). Epub 2021/06/03. doi: 10.3390/ijerph18105194. PubMed PMID: 34068299; PubMed Central PMCID: PMCPMC8153276.

25.	Park JH, Kang SW. College Life Adjustment, Life Stress, Career Barriers, and Career Decision-Making Self-Efficacy of College Students Who Have Experienced COVID-19 in South Korea. Healthcare (Basel, Switzerland). 2022;10(4). Epub 2022/04/24. doi: 10.3390/healthcare10040705. PubMed PMID: 35455882; PubMed Central PMCID: PMCPMC9028693.

26.	Lee H, Park SJ, Kim JE, Kong F, Zhao B, Nam H, et al. International Comparison of Depression During the COVID-19 Pandemic Among University Students in 13 Countries: A Web-Based Cross-Sectional Survey. Asia-Pacific journal of public health. 2021;33(8):928-31. Epub 2021/08/06. doi: 10.1177/10105395211034062. PubMed PMID: 34350800; PubMed Central PMCID: PMCPMC8592100 conflicts of interest with respect to the research, authorship, and/or publication of this article.

27.	Zhao B, Kong F, Nam EW. Assessing Knowledge, Preventive Practices, and Depression among Chinese University Students in Korea and China during the COVID-19 Pandemic: An Online Cross-Sectional Study. Healthcare (Basel, Switzerland). 2021;9(4). Epub 2021/05/01. doi: 10.3390/healthcare9040433. PubMed PMID: 33917674; PubMed Central PMCID: PMCPMC8067962.

28.	Francisco J, Golshan F, Morrison TG, Mickleborough M. Stress and headaches in university students during the COVID-19 pandemic. PLoS One. 2023;18(11):e0288745. Epub 20231122. doi: 10.1371/journal.pone.0288745. PubMed PMID: 37992022; PubMed Central PMCID: PMCPMC10664899.

29.	Wong EL, Li J, Yuen S, Lai AH, Cheung AW, Yau PS, et al. Vulnerable populations during COVID-19 response: Health-related quality of life among Chinese population and its influence due to socio-demographic factors and loneliness. Frontiers in public health. 2022;10:857033. Epub 2022/09/10. doi: 10.3389/fpubh.2022.857033. PubMed PMID: 36081475; PubMed Central PMCID: PMCPMC9446419.

30.	Rathore SS, Lenert LA, Weinfurt KP, Tinoco A, Taleghani CK, Harless W, et al. The effects of patient sex and race on medical students' ratings of quality of life. The American journal of medicine. 2000;108(7):561-6. Epub 2000/05/12. doi: 10.1016/s0002-9343(00)00352-1. PubMed PMID: 10806285.

31.	Ramin F, Aghaei Z, Khorasani FZF. Human love: Physical and mental health from Avicenna's viewpoint. Current problems in cardiology. 2024;49(7):102624. Epub 2024/05/09. doi: 10.1016/j.cpcardiol.2024.102624. PubMed PMID: 38718935.

32.	Esch T, Stefano GB. Love promotes health. Neuro endocrinology letters. 2005;26(3):264-7. Epub 2005/07/02. PubMed PMID: 15990734.

33.	Yu H, Xu S, Li H, Wang X, Sun Q, Wang Y. A man-made divide: Investigating the effect of urban-rural household registration and subjective social status on mental health mediated by loneliness among a large sample of university students in China. Frontiers in psychology. 2022;13:1012393. Epub 2022/12/06. doi: 10.3389/fpsyg.2022.1012393. PubMed PMID: 36467178; PubMed Central PMCID: PMCPMC9712945.

34.	Cao Y, Huang L, Si T, Wang NQ, Qu M, Zhang XY. The role of only-child status in the psychological impact of COVID-19 on mental health of Chinese adolescents. J Affect Disord. 2021;282:316-21. Epub 2021/01/10. doi: 10.1016/j.jad.2020.12.113. PubMed PMID: 33421858; PubMed Central PMCID: PMCPMC8529236.

35.	Ebrahim AH, Dhahi A, Husain MA, Jahrami H. The Psychological Well-Being of University Students amidst COVID-19 Pandemic: Scoping review, systematic review and meta-analysis. Sultan Qaboos University medical journal. 2022;22(2):179-97. Epub 2022/06/09. doi: 10.18295/squmj.6.2021.081. PubMed PMID: 35673293; PubMed Central PMCID: PMCPMC9155030.

36.	Al-Tammemi AB, Akour A, Alfalah L. Is It Just About Physical Health? An Online Cross-Sectional Study Exploring the Psychological Distress Among University Students in Jordan in the Midst of COVID-19 Pandemic. Frontiers in psychology. 2020;11:562213. Epub 2020/11/27. doi: 10.3389/fpsyg.2020.562213. PubMed PMID: 33240151; PubMed Central PMCID: PMCPMC7677563.

37.	Nowak PF, Bożek A, Blukacz M. Physical Activity, Sedentary Behavior, and Quality of Life among University Students. BioMed research international. 2019;2019:9791281. Epub 2020/01/14. doi: 10.1155/2019/9791281. PubMed PMID: 31930143; PubMed Central PMCID: PMCPMC6942714 publication of this paper.

Once again, thanks very much for all your valuable and insightful comments.

Best wishes.

Fanlei Kong

Corresponding Author
