# Supplementary material for: Association between psychological distress, lifestyle and career planning on health-related quality of life among the university students during school closure of COVID-19 pandemic in China
Source: PLoS One. 2026 Apr 30;21(4):e0347032. doi: 10.1371/journal.pone.0347032 (PMC13132209; doi:10.1371/journal.pone.0347032)
Supplement: Attachment — Submitted filename: renamed_132f3.docx [file pone.0347032.s003.docx]

**Revision of ‘PONE-D-25-59759_R1’**

The manuscript has been re-evaluated exclusively with respect to the comments raised in my previous review and considering the authors’ revised submission and response letter. All issues raised in my previous review have been satisfactorily addressed in the revised manuscript.

**Criterion 1: The study presents the results of original research**

In my previous assessment, I noted that although the study appeared to report original findings, the manuscript did not explicitly state that the results had not been published elsewhere. In the revised version, the authors have now clearly stated that the findings have not been published previously and are not under consideration by another journal. This clarification has been incorporated into the manuscript. The concern has therefore been adequately addressed.

**Criterion 2: Results reported have not been published elsewhere**

I previously indicated that the originality of the results should be explicitly declared. The revised manuscript now includes a formal statement confirming that the data and findings are original and unpublished. This issue has been fully resolved.

**Criterion 3: Experiments statistics and analyses are performed to a high standard and are described in sufficient detail**

In the initial review, I acknowledged the appropriate use of validated instruments and statistical models but noted two limitations: the reliance on selected indicators for lifestyle and career planning due to the absence of systematic scales, and the potential representativeness bias introduced by the snowball sampling strategy. In the revised manuscript, the authors have expanded the methodology section, clarified inclusion and exclusion criteria, justified the use of snowball sampling under lockdown conditions, and provided a formal sample size calculation. They have also strengthened the limitations section to explicitly acknowledge potential sampling bias. These clarifications adequately respond to the concerns raised.

**Criterion 4: Conclusions are supported by the data and presented appropriately**

My previous evaluation considered the conclusions to be consistent with the presented results while appropriately acknowledging the cross-sectional design as a limitation for causal inference. The revised version maintains this alignment and continues to recognize the methodological constraints of the study design. No further concerns remain under this criterion.

**Criterion 5: The article is intelligible and written in standard English**

The manuscript was previously considered intelligible and written in acceptable academic English. In the revised version, the authors state that the manuscript has undergone additional language editing by a native English speaker, as acknowledged in the manuscript. The language quality is satisfactory.

**Criterion 6: The research meets ethical standards and is ethically sound**

The original submission included ethical approval information. In the revised version, the authors have restated and clarified the ethics approval and consent procedures, confirming compliance with the Declaration of Helsinki and institutional review requirements. This criterion continues to be met.

**Criteria 7: The article adheres to reporting guidelines and community data availability standards**

Previously, I noted that data sharing was restricted due to privacy concerns. The revised manuscript now clarifies that although the data cannot be publicly shared, anonymized data are available upon reasonable request in accordance with ethical guidelines. This clarification sufficiently addresses the earlier concern.
