# Supplementary material for: Association between psychological distress, lifestyle and career planning on health-related quality of life among the university students during school closure of COVID-19 pandemic in China
Source: PLoS One. 2026 Apr 30;21(4):e0347032. doi: 10.1371/journal.pone.0347032 (PMC13132209; doi:10.1371/journal.pone.0347032)
Supplement: Attachment — Submitted filename: Response to Reviewers.docx [file pone.0347032.s004.docx]

**Point-by-point response to editors and reviewers**

**Authors’ responses to Editor**

Manuscript ID：PONE-D-25-59759

Title：Association between Psychological Distress, Lifestyle and Career Planning on Health-Related Quality of Life among the University Students during School Closure of COVID-19 Pandemic in China

Authors：Baochen Su, Zhengna Sun, Rui Chen, Hui Liu, Xixing Xu, Fanlei Kong

The authors appreciate the editor and reviewers’ kind support on reviewing our manuscript and providing us with insightful and detailed comments. We highly value the editor and reviewers’ thoughtful feedback and the important issues highlighted by them. We have addressed the editor and each reviewers’ comments in a point-by-point response below. For clarity, we have reproduced the reviewers’ original text in full, with “**Q**” representing their comments and “**A**” representing our responses (in red text). Moreover, the authors have highlighted all the modified parts in yellow for the better reading experience by the editor and reviewers.

Below are our point-to-point responses to reviewers' comments:

**Authors’ responses to Academic Author**

**Q1:** Abstract: You need to include the level or strength of the association (e.g., statistical values, strength of relationships, coefficients, etc.) between psychological distress, lifestyle, career planning, and health-related quality of life. Right now, the abstract mentions the association but does not indicate how strong it is.

**A1**: Thank you for your insightful and helpful comments on including the level/strength of the association in the ABSTRACT. This suggestion has been of great help in improving the quality of the manuscript, and the authors attach great importance to it. We have added the level/strength of the association between psychological distress, lifestyle, career planning, and health-related quality of life in the abstract. The revised manuscript is as follow.

**Revised Manuscript (Line 31 – Line 54):**

**Abstract:** The COVID-19 pandemic and associated school closures presented unprecedented challenges to university students' well-being, highlighting an urgent need to understand the factors influencing their health-related quality of life. This study aimed to explore the association between psychological distress, lifestyle, and career planning on the health-related quality of life of university students during the school closure period in China. A cross-sectional study was conducted, collecting data from 1965 Chinese college students locked down in campus during COVID-19 using a snowball sampling method via an online questionnaire platform ([Wenjuan.com](https://wenjuan.com/)). Psychological distress and the health-related quality of life were measured by scales. Descriptive analysis, Chi-squared test and logistic regression analysis were employed to analyze the data. The mean physical component summary score was 47.5, while the mean mental component summary score was 36.8. Students who were women (OR=1.444, *P*=0.003), having a boy or girl friend (OR=1.379, *P*=0.008), with rural hukou (OR=1.446, *P*=0.004), with low psychological distress (OR=4.589, *P<*0.001), high physical activity intensity (OR=3.909, *P<*0.001), a regular studying schedule arrangement (OR=2.553, *P*=0.008), clear career planning (OR=1.570, *P*=0.001) during COVID-19 were more likely to report a good physical component summary. For mental component summary, lower psychological distress (OR=8.330, *P*<0.001), a regular studying schedule arrangement (OR=2.892, *P*=0.001) and keeping same job-hunting pressure (OR=1.852, *P*=0.003) were positive influencing factors, whereas having a boy or girl friend (OR=0.774, *P*=0.032) and having no clear career planning (OR=0.752, *P*=0.020) during COVID-19 were negative influencing factors. In conclusion, while health-related quality of life was generally good, lower psychological distress, healthier lifestyle habits, and clearer career planning were significantly associated with better health-related quality of life among university students during the pandemic-related school closure. These findings underscore the importance of integrating mental health support, lifestyle guidance, and career planning services into student support systems during public health crises. Future studies should develop and evaluate interventions targeting these modifiable factors.

**Q2:** Conclusion: The conclusion is considered confusing and should more clearly reflect the original aim of the study. It needs to align directly with what the research set out to investigate.

**A2:** The authors greatly appreciate the editor’s constructive and valuable comments on the CONCLUSION. Following the editor’s important suggestion, we have carefully revised the conclusion to make it clearer. The revised conclusion now directly aligns with the original aim of the study and explicitly reflects the key issues that this research intended to investigate. The revised manuscript is as followed.

**Revised Manuscript (Line 349 – Line 365):**

~~In summary, to the best of our knowledge, this was the first study to explore the determinants of HRQOL among Chinese university students during the school closure of the COVID-19 from the perspective of psychological distress, lifestyle habits and life planning.~~ Lower psychological distress, better lifestyle and clearer career planning were significantly associated with the better HRQOL of university students during the school closure of the COVID-19. Maintaining a regular studying schedule arrangement and a relatively high-intensity of exercise during the school closure of the COVID-19 may have a good impact on maintaining a good HRQOL of university students. Intervention measures derived from this research could be put into practice for governments and universities in the future public health emergencies.

Once again, thanks very much for all your valuable and insightful comments.

Best wishes.

Fanlei Kong

Corresponding Author

**Authors’ responses to Reviewer 1**

**Q1:** The manuscript has been re-evaluated exclusively with respect to the comments raised in my previous review and considering the authors’ revised submission and response letter. All issues raised in my previous review have been satisfactorily addressed in the revised manuscript.

**A1:** Thank you very much for the valuable comments you provided during the review process. Your feedback has played a significant role in improving the quality of this paper. Once again, thank you for your help and support.

Once again, thanks very much for all your valuable and insightful comments.

Best wishes.

Fanlei Kong

Corresponding Author

**Authors’ responses to Reviewer 2**

**Q1:** The abstract summarises the work, giving the association between psychological distress, lifestyle and career planning on the health-related QoL of university students, but failed to include the level of association in the abstract.

**A1:** The authors are very honored to receive your insightful review and thank you for your constructive and valuable comments. Recognizing the importance of these points, we have added the coefficient of the association between psychological distress, lifestyle, career planning, and health-related quality of life in the abstract. The revised manuscript is as followed.

**Revised Manuscript (Line 31 – Line 54):**

**Abstract:** The COVID-19 pandemic and associated school closures presented unprecedented challenges to university students' well-being, highlighting an urgent need to understand the factors influencing their health-related quality of life. This study aimed to explore the association between psychological distress, lifestyle, and career planning on the health-related quality of life of university students during the school closure period in China. A cross-sectional study was conducted, collecting data from 1965 Chinese college students locked down in campus during COVID-19 using a snowball sampling method via an online questionnaire platform ([Wenjuan.com](https://wenjuan.com/)). Psychological distress and the health-related quality of life were measured by scales. Descriptive analysis, Chi-squared test and logistic regression analysis were employed to analyze the data. The mean physical component summary score was 47.5, while the mean mental component summary score was 36.8. Students who were women (OR=1.444, *P*=0.003), having a boy or girl friend (OR=1.379, *P*=0.008), with rural hukou (OR=1.446, *P*=0.004), with low psychological distress (OR=4.589, *P<*0.001), high physical activity intensity (OR=3.909, *P<*0.001), a regular studying schedule arrangement (OR=2.553, *P*=0.008), clear career planning (OR=1.570, *P*=0.001) during COVID-19 were more likely to report a good physical component summary. For mental component summary, lower psychological distress (OR=8.330, *P*<0.001), a regular studying schedule arrangement (OR=2.892, *P*=0.001) and keeping same job-hunting pressure (OR=1.852, *P*=0.003) were positive influencing factors, whereas having a boy or girl friend (OR=0.774, *P*=0.032) and having no clear career planning (OR=0.752, *P*=0.020) during COVID-19 were negative influencing factors. In conclusion, while health-related quality of life was generally good, lower psychological distress, healthier lifestyle habits, and clearer career planning were significantly associated with better health-related quality of life among university students during the pandemic-related school closure. These findings underscore the importance of integrating mental health support, lifestyle guidance, and career planning services into student support systems during public health crises. Future studies should develop and evaluate interventions targeting these modifiable factors.

**Q2:** The main paper introduces COVID-19, the students and their demographic factors, as well as the health-related quality of life. They further show an account of the reviewed literature. The methods are also well explained. The results and discussion were also well written, but the conclusion was confusing. It should reflect what they aimed to do.

**A2:** The authors sincerely appreciate the reviewer for the positive feedback and constructive comments on our manuscript. We are grateful that the introduction, literature review, methodology, results, and discussion were recognized as clear and well‑presented.

Following the reviewer’s valuable suggestion, the authors have thoroughly revised the CONCLUSION section. The revised parts now directly corresponds to the original aims of the study and clearly summarizes the key findings in line with what the research intended to investigate. The revised manuscript is as followed.

**Revised Manuscript (Line 349 – Line 365):**

~~In summary, to the best of our knowledge, this was the first study to explore the determinants of HRQOL among Chinese university students during the school closure of the COVID-19 from the perspective of psychological distress, lifestyle habits and life planning.~~ Lower psychological distress, better lifestyle and clearer career planning were significantly associated with the better HRQOL of university students during the school closure of the COVID-19. Maintaining a regular studying schedule arrangement and a relatively high-intensity of exercise during the school closure of the COVID-19 may have a good impact on maintaining a good HRQOL of university students. Intervention measures derived from this research could be put into practice for governments and universities in the future public health emergencies.

Once again, thanks very much for all your valuable and insightful comments.

Best wishes.

Fanlei Kong

Corresponding Author
